# Evidence-based malaria control in Timor Leste from 2006 to 2012

**DOI:** 10.1186/s12936-015-0614-6

**Published:** 2015-03-11

**Authors:** Manel AMG Yapabandara, Raul Sarmento, Maria do Rosario de Fatima Mota, Johanes don Bosco, Nelson Martins, Ananda R Wickremasinghe

**Affiliations:** Formerly World Health Organization, Dili, Timor Leste; Ministry of Health, Dili, Timor Leste; University of Timor Leste, Dili, Timor Leste; Department of Public Health, Faculty of Medicine, University of Kelaniya, Ragama, Sri Lanka

**Keywords:** Timor Leste, Evidence based malaria control, RDTs, LLINs, Entomological surveillance, Quality assurance of microscopy

## Abstract

**Background:**

Malaria has been a major public health problem in the newly established Democratic Republic of Timor Leste with over 200,000 cases being reported in 2006 and 2007. The National Malaria Control Programme (NMCP) was established in 2003. The progress made in malaria control in Timor Leste is reported.

**Methods:**

Records maintained at the NMCP, the district health services, the Health Information and Management System, the National Laboratory on malaria diagnosis and entomological data of the NMCP were reviewed.

**Results:**

There has been a 97% decrease in the reported malaria incidence from 2006 (223,002 cases) to 2012 (6,202 cases). 185,106 clinical cases reported in 2006 decreased to 2,016 in 2012 with introduction and expansion of malaria microscopy services and introduction of monovalent RDTs in 2008 and bivalent RDTs in 2010 in all parts of the country. The National Treatment Guidelines using ACT as the first-line treatment for *Plasmodium falciparum* infections and introduction of monovalent RDTs, led to a 42% and a 33% decrease from 2007 to 2008 in reported clinical and total malaria cases, respectively. LLINs were distributed initially to pregnant females and children under five and later per every two persons living in high-risk areas (based on microstratification at sub-district level). IRS was carried out in three districts in 2010 and extended to six districts in 2012. *Anopheles barbirostris* and *Anopheles subpictus* have been incriminated as malaria vectors. A National Laboratory, which routinely cross checks blood smears for quality assurance of microscopy was established. Malaria focal points at regional, district and sub district level, entomology surveillance staff, monitoring and evaluation officers, and quality control technicians were appointed to strengthen malaria control activities at all levels in the country.

**Conclusion:**

The 97% decrease in the incidence of malaria in Timor Leste is due to application of evidence-based malaria control methods that included enhancing improved quality surveillance, early diagnosis and prompt treatment of cases with effective anti-malarials, targeted vector control, human resource development and deployment, commitment of staff, GFATM funding and technical assistance from WHO.

## Background

Malaria is endemic to 104 countries of the world [1]. The burden of malaria has decreased significantly during the last decade. There were an estimated 207 million cases of malaria (range 135–287 million) and 627,000 malaria deaths (range 473,000–789,000) in 2012 [1]. Of the 104 malaria endemic countries, 52 countries are on track to meet the Roll Back Malaria Initiative target to reduce malaria case incidence by 75% of the 1990 figure by 2015; twelve countries were in the pre-elimination phase, seven were in the elimination phase and seven were in the prevention of re-introduction of malaria phase by 2012 [1].

Malaria is prevalent in 10 out of the 11 countries of the WHO Southeast Asian region. The Maldives successfully eliminated indigenous malaria in 1984. No indigenous case of malaria has been reported in Sri Lanka since October 2012. Malaria re-emerged in the Democratic Republic of Korea in 1997/98. About 40% of the global population at risk of malaria resides in the Southeast Asia region which accounts for about 15% of global reported confirmed malaria cases and around 2.7% of the global mortality due to malaria [2]

Malaria has always been a major public health problem throughout Timor Leste [3]. The exact distribution of malaria has not been documented. There have been reports to suggest that transmission tends to be mesoendemic on the coast, hypoendemic in the inland lowland regions, and very low to absent in areas over 500 m above sea level [4,5]. Malaria transmission peaks in Timor Leste from January to April after the rainy season [6].

The National Malaria Control Programme (NMCP) was established in 2003. Prior to 2005, limited interventions were carried out supported by The Global Fund fight against AIDS, Tuberculosis and Malaria (GFATM) Round 2 [7,8] and other non-governmental and aid agencies. Organized malaria control activities were initiated after 2006. The implementation of organized evidence based malaria control methods that have led to a 97% decrease in the incidence of malaria since 2005 is documented here.

## Methods

### The country

Timor Leste, (formerly East Timor) occupies the eastern part of Timor, the largest island at the eastern end of the Lesser Sunda Islands which form part of the Malayan Archipelago, the nearby islands of Atauro and Jaco, and Oecussi, an exclave in Indonesias’s West Timor [9]. Timor Leste gained independence from Indonesia in 2002. The island of Timor is located between 8°50′S and 125°55′E; the country occupies a land area of 15,007 square kilometers [10]. The island topography consists of 80% mountains; the coastal plains are narrow swamp with no major highland valleys or permanent rivers. The country is divided into 13 districts and 65 sub-districts. The estimated population for 2013 was 1.18 million.

### Health system

The delivery of health care services is under the Ministry of Health. The Ministry of Health has a Director General who is in charge of implementing all policies and programmes. There are five National Directors under the Director General for Planning and Finance, Community Health, Logistics and Procurement, Human Resources and Hospital Services. The Communicable Diseases Control (CDC) directorate, of which the NMCP is a part of, is under the National Director for Community Health [11].

Curative services are provided through a network of health posts at the grass roots level. In general, health posts serve communities of about 1,000 population. Health posts come directly under community health centres (CHC), which are sub-district level institutions. Thirteen CHCs have inpatient facilities and are managed by qualified doctors. Currently, there are about 135 health posts and 67 CHCs. In addition, there are five referral hospitals located in different districts and a national hospital located in the capital city of Dili. There are community health specialty volunteers who diagnose and treat uncomplicated malaria patients living in rural, hard to reach villages except pregnant women and infants under 1 year.

There is an extensive well-organized community health volunteer network attached to the Ministry of Health. These volunteers assist the NMCP in malaria prevention activities. There are also private health care facilities situated in the main towns. Traditional healers and practitioners are also popular.

### National malaria control programme

The NMCP was established in 2003 under the Communicable Disease Control Department of the Ministry of Health. At its inception in 2003, the NMCP employed two temporary national malaria control officers and one driver funded by the GFATM round 2 Grant. The NMCP was responsible for planning, implementation, monitoring and evaluation of malaria control activities in the country. The district health teams were responsible for district health planning, coordination of health service delivery, management of logistics, and monitoring and evaluation. At the district level, one district malaria public health officer (DPHO) attached to each district was responsible for control of all communicable diseases until 2009. With funding from the GFATM round 7 grant in 2009, a staff of 43 dedicated for malaria control including regional malaria officers, entomology and vector control assistants, insect collectors, data entry assistants, quality control malaria microscopy technicians at the national level and 13 district malaria officers and 28 malaria assistants in high risk sub-districts were recruited.

Currently, after implementation of the GFATM round 10 grant programme, the NMCP has 161 staff at central, district and sub-district levels, cohesively functioning with the health management team. The Ministry of Health provides infrastructure and human resources for diagnosis and treatment of malaria cases. The major source of funding for the NMCP since 2003 has been the GFATM. The World Health Organization provided technical assistance for the NMCP on a short-term basis since 2002 and on a long-term basis since 2009 for planning, monitoring and evaluation, and implementation of the NMCP.

The overall goal of the NMCP is to reduce the burden of malaria in Timor Leste in line with the global malaria control strategy adopted in 1992 by a Ministerial Conference on Malaria held in Amsterdam, and subsequently endorsed by the World Health Assembly and the UN General Assembly in 1993 and in line with WHO-SEARO’s scaled-up strategy. The strategy comprises six imperative strategic approaches; 1) Enhancing case management through early case detection and delivery of effective anti malarial therapies, 2) Distribution of long-lasting insecticidal nets (LLINs) targeting vulnerable populations (initially to children below five years of age and pregnant women, which was later modified to one LLIN per two persons living in malaria risk areas), 3) Integrated vector management using indoor residual spraying (IRS), environmental manipulation, larval control, and personal protection, 4) Epidemic/outbreak prevention, preparedness and response, 5) Behavioural change communication (BCC) through information, education and communication (IEC) campaigns, and 6) Enhancing monitoring, evaluation and operational research.

Entomological surveillance in the districts was carried out by the NMCP for the establishment of an evidence based vector control programme. Furthermore, quality control of malaria microscopy was established in the National Laboratory; a sample of blood smears and RDTs collected every month from the medical laboratory analysts attached to CHCs and hospitals are sent to the National Laboratory for cross checking.

### Data collection

Data were collected from the NMCP office in Dili, Timor Leste, the district health services and the health information and management system. Routinely collected monthly data on malaria incidence were available district wise since 2005. Since 2009, routine data collection systems have been in place and a formal verification system was introduced as part of the requirements of the GFATM, the major funding source of the NMCP. Relevant documents were checked to verify events that are described here.

## Results and discussion

Malaria is prevalent throughout Timor Leste and is perennial. The incidence of malaria has decreased from a high of about 223,000 cases in 2006 to 6148 cases in 2012, a 97% decrease within a seven-year period (Table 1). Based on data from 2006, the major transmission season extends from January to about April corresponding to the rainy season. In some years, there has been a minor peak from May to July. Both *Plasmodium falciparum* and *Plasmodium vivax* are prevalent in the country. *Plasmodium falciparum* is the predominant species, generally accounting for over 70% of confirmed cases except in 2011 and 2012 when the incidence was low.

**Table 1:** **Monthly Malaria incidence (clinical and confirmed cases) 2006-2012**

**Month**	**2006**	**2007**	**2008**	**2009**	**2010**	**2011**	**2012**
January	24,051	23,726	18,007	21,873	11,200	6,586	969
February	27,017	28,470	15,397	15,122	13,413	5,783	948
March	30,842	22,826	11,374	14,316	15,265	4,790	787
April	23,381	22,050	14,621	14,147	9,522	4,104	504
May	17,260	15,601	11,695	9,288	10,564	4,675	455
June	15,709	14,679	14,752	8,574	9,732	3,004	594
July	15,523	17,639	11,568	7,074	8,271	2,061	340
August	13,748	13,276	7,884	8,147	7,085	1,472	531
September	14,077	15,426	8,553	9,173	7,043	1,220	370
October	12,912	14,470	8,749	11,385	10,767	1,092	330
November	13,410	13,450	9,219	7,446	11,463	760	201
December	15,072	13,789	11,775	6,584	5,133	572	119
**Total**	**223,002**	**154,438**	**215,402**	**143,594**	**119,458**	**36,119**	**6,148**

The NMCP has carried out entomological surveys since 2006 for the implementation of an evidence based vector control programme in the country. Regular entomological surveillance has been carried out since 2006; 13, namely *Anopheles aconitus*, *Anopheles barbirostris*, *Anopheles subpictus*, *Anopheles sundaicus*, *Anopheles varuna*, *Anopheles maculatus*, *Anopheles minimus*, *Anopheles vagus*, *Anopheles peditaeniatus*, *Anopheles annularis*, *Anopheles tessellatus* and *Anopheles kochi* have been identified*.* Previously Cooper *et al*. had reported seven Anopheline species; *An. barbirostris, An. aconitus, An. annularis, An. maculatus, An. peditaeniatus, An. sundaicus* and *An. vagus* based on a survey carried out from February to June 2001 comprising 2030 samples [9]; *An. barbirostris* and *An. vagus* genotype B were then incriminated as malarial vectors.

Based on the sporozoite detection by salivary gland dissection and ELISA, *An. barbirostris* and *An. subpictus* have been incriminated as vectors of malaria in the country [6]; the entomological inoculation rates of these species were 3.16 and 1.75 infective bites per person per year, respectively. These vector species rest mainly indoors and demonstrated early biting behaviour (18:00 to 2:00 hrs). The peak biting time of *An. barbirostris* is from 22:00 to 02:00 hrs. The outdoor:indoor human biting ratio of *An. barbirostris* and *An. subpictus* was 1:1.5 respectively. Furthermore both species rest on walls, under furniture and roofs till maturation of eggs. *An. barbirostris* mainly breeds in streams and rivers with vegetation, in paddy fields and in rain water collections. *Anopheles subpictus* breeds in fresh and brackish water marshy lands, and in river and stream bed pools. Insecticide susceptibility test results carried out since 2007 indicated that both species are susceptible to DDT 4%, malathion 5%, deltamethrin 0.05%, permethrin 0.75%, alfacypermethrin, lambdacyhalothrin 0.05%, bifenthrin 0.2%, bendiocarb 0.1%, fenitrothion 1%, cyflothrin 0.15% and carbamates, Based on the results of entomological surveys, it was decided to carry out indoor residual spraying using lambdacyhalothrin in the epidemic prone malaria high risk areas. As the vectors were susceptible to pyrethroids and the peak biting time of the major vector coincides with the sleeping pattern of people, it was planned to distribute LLINs with pyrethroids.

Many reasons may be attributed to the reduction in malaria incidence from 2006 to 2012. As expected, there has been a gradual reduction in the number of cases of clinical malaria with enhancement of diagnostic facilities (Table 2). The population protected against malaria (Table 2) and recruitment of staff as focal points at national, district and sub-district level have also increased over the years. The different strategies that resulted in these improvements are documented and discussed.

**Table 2:** **Malaria cases and malaria control methods used in Timor Leste 2006-2012**

**Indicator**	**2006**	**2007**	**2008**	**2009**	**2010**	**2011**	**2012**
Population	1,015,187	1,047,632	1,080,742	1,114,534	1,149,028	1,092,104	1,118,429
Clinical malaria cases	185.106	168,533	97,621	85,799	70,969	16,418	940
Confirmed Pf cases	24,219	34,174	34,406	34,517	36,528	14,261	2,016
Confirmed Pv cases	13,477	12,544	11,296	12,246	11,492	3,759	2,288
Confirmed mixed cases	200	151	272	567	469	1,720	958
Total cases	223,002	215,402	143,594	133,139	119,458	36,153	6,202
% change from previous year	-	-3.4	-33.4	-7.3	-10.3	-69.7	-82.8
% pf	64.5	73.2	73.2	74.1	76.3	81.0	56.5
Malarial deaths	58	26	10	56	58	16	4
Incidence/1000 population	220	206	133	119	104	33	6
Blood smears taken	96,485	114,283	92,870	85,538	110,494	82,175	64,318
RDTs used	0	0	0	34,152	85,751	127,272	117,599
LLINs distributed*	58,153	97,704	79,226	-	170,985	23,493	25,143
Houses sprayed with IRS	-	-	-	-	12,751	16,888	27,228
population protected with LLINs and IRS	116,306	311,714	470,166	353,860	500,422	388,956	219,621

*Life expectancy of LLINs considered as three years.

Since the establishment of the NMCP in 2003, many improvements and advances have taken place. In the early years, the NMCP was dependent on other agencies for malaria control due to a severe shortage of staff, and lack of infrastructure, technical expertise and funding. Even though funding from GFATM was obtained in 2003, the NMCP relied heavily on other partners and agencies that were contracted as sub-recipients to carry out malaria control activities. During these years, there was no estimate of the burden of malarial disease or a reliable data reporting system. A large number of malaria cases were detected clinically and facilities for microscopy were limited.

**Figure 1:**
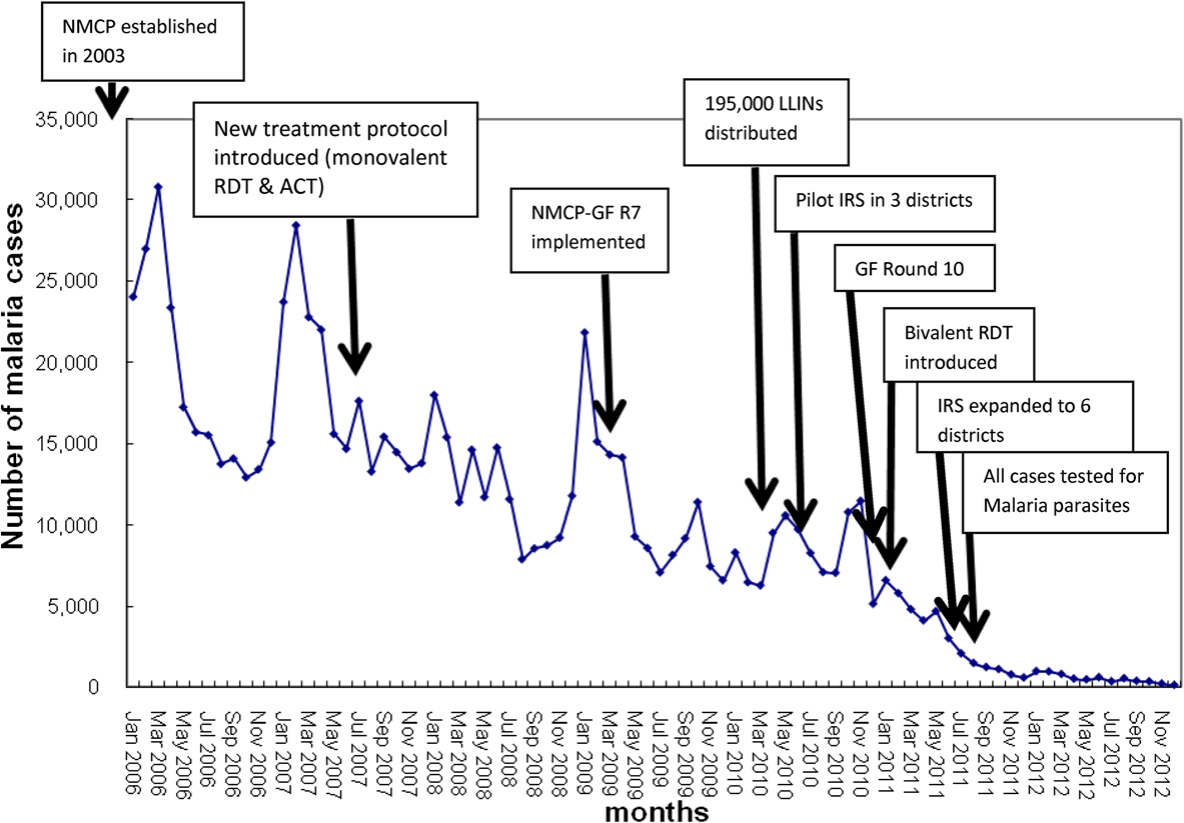
**Number of malaria cases in Timor Leste 2006-2012.**

Subsequently, the NMCP developed a comprehensive malaria control programme with assistance from WHO. In 2006, the national malaria control strategy 2005-2013, in conformity to the 1992 global strategy on malaria control, was implemented (Figure 1). Furthermore, this strategy was revised in 2009 with assistance from WHO. The strategy was based on microstratification at sub-district level based on malaria receptivity data of 2008.

**Figure 2:**
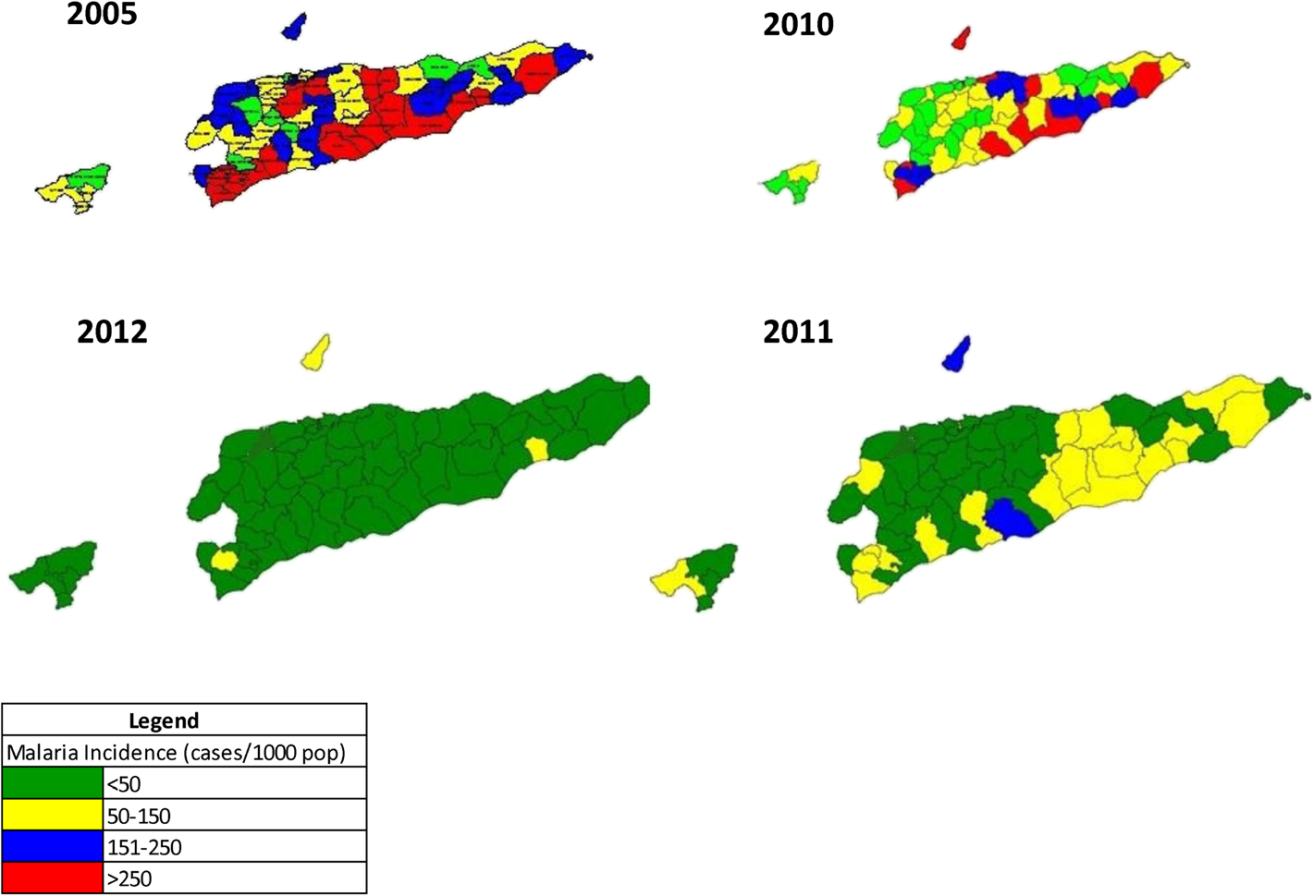
**Microstratification of sub-districts based on malaria incidence.**

Microstratification of sub-districts was carried out based on the incidence of malaria (IM), the annual parasitic incidence (API), annual rainfall and rainfall duration, elevation from mean sea level, breeding sources and their extent, number of paddy cultivation areas and seasons and the abundance of vector species. The sub-districts were grouped as high-risk areas, moderate risk areas and low risk areas. The criteria used for classification is given in Table 3. A sub-district had to satisfy at least three criteria to be considered in a particular category. Based on the miscrostratification using 2005 data, 37 sub-districts were classified as high-risk areas, 16 as moderate risk areas and 8 as low risk areas (Figure 2).

**Table 3:** **Criteria used for microstratification of subdistricts**

**Criterion**	**High risk area**	**Moderate risk areas**	**Low risk areas**
Malaria incidence (per 1000 population)	≥150	50-149	<50
Annual Parasite Incidence (per 1000 population based on confirmed malaria cases)	>35	25-34	<25
Geographic area	Southern coastal areas	Northern coastal areas	Central forest areas (between southern and northern areas)
Elevation (meters)	0-500	0-1000	1000-2500
Annual rainfall (mm)	1000-1500	<1000	>1500
Rice cultivation seasons per year	2	1	-
Vectors	*An. barbirostris An. subpictus*	*An. barbirostris An. subpictus*	*An. barbirostris An. subpictus*

With the microstratification of sub-districts, control strategies and activities for different risk areas were planned (Table 4). Although these strategies and activities were planned in 2007, organized implementation of these strategies was done only in 2009 and thereafter with funding available from the GFATM and technical guidance from the WHO.

**Table 4:** **Malaria control activities implemented by risk stratification**

**Activity/Method**	**High risk areas**	**Moderate risk areas**	**Low risk areas**
**Early diagnosis and prompt treatment**			
Activated Passive Case Detection	Yes	Yes	Yes
Active Case Detection	Yes	Yes	Yes
Outreach clinics	Yes	Yes	Yes
**Vector control**			
LLINs (pregnant women)	Yes	Yes	Yes
LLINs (1 per 2 persons)	Yes	Yes	In selected areas
Indoor Residual Spraying	Selected areas	Epidemic prone areas	During epidemics
Larvivorous fish	Yes	Yes	Yes
Environmental manipulation/modification	Yes	Yes	Yes
Chemical larviciding	Yes	Yes	Yes
Personal protection	Yes	Yes	Yes
Behavioural change communication	Yes	Yes	Yes

The main focus of the strategy was to enhance routine diagnosis and treatment of cases, and to implement selected control methods as and when needed and available. A national quality control laboratory was set up to train microscopists and to cross check blood smears. Microscopists were trained in detecting malaria parasites on Giemsa-stained thick and thin blood smears. Furthermore, rapid diagnostic test kits (RDT), which detect *P. falciparum,* was introduced to health posts with no malaria microscopists and emergency services of hospitals in 2007. Monovalent RDTs that detect *P. falciparum* and non-*P. falciparum* were introduced to all health facilities along with microscopy and to the health posts where there were no microscopists. Health staff was trained on use of RDTs.

In 2007, the national malaria treatment guidelines were implemented. These guidelines resulted in the introduction of monovalent RDTs and artemisinin combination therapy (ACT) for falciparum malaria. The adoption of these guidelines resulted in a 33% decrease in the incidence of clinical malaria cases that were treated based on a clinical diagnosis without laboratory confirmation and in the total malaria cases in 2008 as compared to 2007. There was a doubling of the number of persons tested for malaria parasites in subsequent years with the introduction of RDTs. ACT was made available at all government health facilities in all districts and health care staff were educated about the use of ACT. The new treatment guidelines introducing RDTs and ACT would have contributed to a significant decrease in the reservoir of infection with elimination of drug resistant strains. Prior to the introduction of ACT, sulphadoxine-pyrimethamine (SP) was used to treat falciparum infections. The therapeutic efficacy tests carried out by Burns *et al.*, revealed that 80% of the *P. falciparum* population was resistant to SP [12]. All *P. vivax* cases were treated with chloroquine and 14 days of primaquine. There was a drastic reduction of malaria after introduction of bivalent RDTs in 2011 and expansion of malaria microscopy services. In addition, all health staff were advised that malaria treatment should be carried out only on a confirmed diagnosis. Specially selected community health volunteers were appointed to hard to reach malaria risk areas for diagnosis and treatment of uncomplicated malaria.

As a consequence to the unexpected dramatic reduction in the incidence of malaria in 2012, large quantities of outdated drugs that were procured expecting a much higher incidence of malaria had to be destroyed. Despite this loss, which may be considered “wastage” in economic terms, the overall improvement in the well-being of the population far outweighs the loss, when factors such as debilitation, and loss of income are considered.

The introduction of bivalent RDTs in 2011 and its scaling up resulted in an 80% decrease in the number of clinical cases and a 69% decrease in the number of total malaria cases reported in 2011 as compared to 2010. Diagnostic facilities were made available at all government treatment facilities including health posts in all districts of the country. All workers in health institutions were trained on use of bivalent RDTs. In addition, all clinicians and clinical nurses attached to health institutions were trained on implementation of the national malaria treatment protocol. Health workers attached to private health clinics were educated on the national malaria treatment protocol. Health staff attached to selected faith based organizations and private clinics were trained on the use of malaria microscopy and RDTs and the National Malaria Treatment Protocol. The Ministry of Health implemented a programme of integrated community health assistance called “ServisuIntegradu da SaúdeCommunitária or Integrated Community Health Services (SISCa)” with the objective of providing integrated health assistance to the smallest community units (aldeias) to ensure that every community has access to such a service. Malaria diagnosis and treatment, and health education were carried out in hard to reach areas during the CISCa mobile clinics. By August 2011, there were very few clinical malaria cases reported as almost all suspected cases were examined for malaria parasites either by microscopy or by RDTs.

WHO prequalified anti-malarials were procured and stocks at the national, district and health facility level were monitored. It was observed that there was no stock out more than 7 days from 2009 to 2012. The improvement in diagnostic facilities, availability of quality drugs, no stock out situation, and training of clinicians resulted in a 70% reduction in malaria mortality as well.

All positive blood smears and 10% of negative blood smears taken by laboratory analysts were cross-checked at the national laboratory and feedback was sent to the respective laboratory analysts. A database was developed at the national referral laboratory to monitor the performance of laboratory analysts. All laboratory analysts who had an agreement rate of less than 90% with a quality control laboratory analyst were given further training at the national level or as on the job training to improve their performance. The majority of laboratory analysts were given refresher training on malaria microscopy annually. Quality control of RDTs was carried out at the “SAMES” (ServiçoAutônomo de MedicamentosEquipamentos de Saúde-drug stores) at hospital, community centre, health post and mobile clinic levels. The improvement of malaria diagnostic facilities indirectly improved delivery of health care services in general. Having a definite diagnosis of a positive or a negative result for malaria permits the practicing clinician to focus on appropriate treatment. Studies done to assign malaria as a cause of death in children based on a clinical diagnosis, using data from the health management information system of the Ministry of Health, have reported sensitivities ranging from 45% to 72% though specificities have been higher than 85%. The same may be applied to treatment based on a clinical diagnosis. Excluding malaria is also important as its presentation is similar to many other infectious diseases.

The commencement of the NMCP GFATM Round 7 project in April 2009 signaled a significant landmark in malaria control in Timor Leste. Based on insecticide susceptibility test results, the biting behavior of the *An. barbirostris* and the capacity of the NMCP it was decided to protect pregnant women and children under 5, categorized as malaria high-risk groups, through the distribution of LLINs. Initially, based on microstratification, LLINs were distributed to pregnant women and children under five in the risk districts. With funding available from the GFATM, 195,000 LLINs were procured and distributed commencing April 2010, protecting 90% of children under five in high-risk areas from infective malaria bites. Later, in 2010, the policy was changed and one LLIN was distributed per every two persons in malaria risk areas. Simultaneously, bioassay testing was commenced using primarily wild caught *An. barbirostris* and *An. subpictus* species to monitor the persistence of insecticides in the fabric of LLINs as part of quality control of LLINs

In August 2010, IRS using lambdacyhalothrin 0.05% WP was carried out as a pilot project in selected areas in three districts by the district health services in collaboration with community health volunteers and community leaders to supplement the LLIN effect. 12,751 houses in epidemic prone high-risk malaria villages were fully sprayed with 10% WP lambdacyhalothrin achieving a coverage of 96% of targeted houses and protecting 86,253 population. IRS was carried out in selected areas as a pilot study to evaluate the feasibility of carrying out IRS in the country before expanding IRS into high-risk areas of other districts. Based on the successful implementation of the pilot study, IRS was expanded into 6 malaria risk districts; in 2011 and 2012, 10% WP lambdacyhalothrin was used to spray 16,888 and 27,228 houses protecting 102, 858 and 105,743 population living in epidemic prone areas, respectively. Quality of IRS was assessed by detection of persistence of insecticide on sprayed surfaces; quality of spraying of the spray machine operators was assessed by bio-assay testing in randomly selected sprayed houses. Furthermore, spraying was supervised to ensure use of high quality spraying technique, high spray coverage and to reduce pilferage of insecticides. Bendiocarb WP, which is chemically unrelated to pyrethroids will be used for IRS in 2015 in order to reduce the pressure on pyrethroids as more than 80% of the malaria risk population will be protected by pyrethroid-treated LLINs by the end of 2014 to minimize the chances of emergence of pyrethroid resistance in malarial vectors.

Behavioral change communication is important to improve or initiate favorable behaviours towards malaria prevention and control by the community as well as the health staff. Knowledge of, attitude towards and practices related to malaria were investigated in the malaria indicator sample (MIS) Survey carried out in 2010. Based on the findings of the MIS, health promotion and education of the community was carried out using group discussions, home visits, dramas enacted by district health teams with assistance of community health volunteers and GFATM sub recipient, Health Net Timor Leste. 123,354 and 237,008 persons living in malarious areas were educated on malaria prevention and control in 2011 and 2012, respectively.

A dramatic 97% decline in the incidence of malaria in Timor Leste in 2012 as compared to 2006 is documented here. The decrease in the incidence can be attributed to evidence-based strategies that were implemented at different points in time. The reduction is a combination of the individual and synergistic effects of many strategies. Further analyses need to be done to determine which of the strategies is the most cost effective.

Current malaria incidence data suggest that Timor Leste may consider to progress to malaria pre-elimination and elimination phases. This will take much commitment as the major hindrance to achieve such a status will the common border it shares with West Timor of the Republic of Indonesia. It will be a significant achievement if both West Timor and Timor Leste work in unison to achieve this goal, which is realistic given the achievements documented here.

Based on the recommendations of the external expert review panel of the NMCP in 2013, the NMCP in Timor Leste proposes to align the programme towards malaria pre-elimination targets at subnational level. An increasing number of subdistricts are reaching pre-elimination status; stratification of areas needs to be revised based on current API (<0-1, 1.1-10, >10.1) with setting up of specific interventions to interrupt indigenous transmission in these areas. Consolidating the remarkable success is the immediate priority in a still fragile environment (health and political wise) while malaria has dropped out of the top five diseases in the country. Investments in malaria control have to continue to maintain gains by supporting essential interventions such as maintaining universal LLIN coverage, strengthening surveillance in remote areas, ensuring continued access of the population to quality diagnosis and treatment and encouraging dedicated skilled staff to perform their duties optimally. Such investments in malaria control will accrue many indirect benefits to the country including the tourist industry and foreign direct investments for human development.

The extraordinary gains in malaria control that have been achieved thus far in Timor Leste has been through the implementation of an effective programme dedicated to malaria control in coordination with a health system that is undergoing reform. At this stage of malaria control, it is mandatory that control activities focus operations through a mechanism that is dedicated for malaria control in the next few years. To sustain gains in a proposed decentralized context, the programme by its own initiative and processes should focus to enhance and sustain the following interventions during the coming years: (1) data management, surveillance and outbreak response in alliance with the HMIS, (2) case management with quality diagnosis to ensure treatment is based on evidence of a confirmed diagnosis, (3) entomological surveillance and vector control operations such as well supervised IRS in remaining high endemic areas (where API is more than 10/1,000) and flood affected epidemic prone areas coupled with LLIN coverage, (4) revision of the malaria receptivity micro stratification criteria based on an API <1, 1.1-10, >10.1 per 1,000 population as the incidence of malaria reduced to six per 1,000 population in 2012 (5) universal LLIN coverage of all endemic areas with an API >1/1000 (6) enhancing capacity of the NMCP through progressive recruitment of skilled staff by the government of Timor Leste, (7) improved collaboration with other departments within the Ministry of Health towards more integration of services adopting a primary health care approach, (8) inventory control and forecasting of anti-malarial drugs, RDTs and equipment, and stock management within the programme until the national procurement and supply chain management systems are well established and functionally operational, (9) ensuring regulation and quality assurance of anti-malarial drugs through regulation of drug imports until the Drug Regulatory Authority is functionally operational and updating of essential drug list excluding artemisinin monotherapies, (10) increasing collaboration with private companies that attract migrant workers in Timor Leste to prevent introduction of indigenous malaria transmission from imported malaria cases, (11) strengthening cross-border collaboration to mitigate epidemics due to illegal migration of workers and to contribute to set up a broad migrant policy, (12) strengthening community engagement in surveillance and personal protection including recognition and control of potential breeding sites to maintain receptivity at low levels, (13) ensuring availability of long-term malaria technical expertise, and (14) strengthening intersectoral collaboration with other ministries such as the Ministries of Agriculture and Fisheries, and Infrastructure and Public Administration for implementation of pesticide law, risk mitigation, and to delay the emergence of insecticide resistance as part of the integrated pest and vector management strategy adopted by the NMCP with farmer/farmer family participation who live in rice cultivation malaria risk areas for sustainable malaria control and prevention.

The major contribution to this achievement has been the commitment of the officers of the NMCP and the government, generous funding provided by the GFATM and technical guidance from the WHO. GFATM funds have been mostly responsible for the planning and implementation of the NMCP in Timor Leste up to now. The government of Timor Leste is now demonstrating more commitment in allocation of more resources towards malaria control largely in terms of providing dedicated clinical and laboratory staff for the programme, recruiting 13 district malaria officers funded by the Global Fund as permanent officers funded by MOH, procurement of insecticides for Indoor Residual Spraying and Long Lasting Insecticide treated net distribution. However, still much more commitment is required in the future to ensure that the programme is sustained at the same level as it is now especially after when the GFATM grant will end. Sustaining the NMCP that has made significant achievements since its inception barely 10 years ago is a national priority.
